# Clinical characteristics and risk factors for culture-negative periprosthetic joint infections

**DOI:** 10.1186/s13018-021-02450-1

**Published:** 2021-05-03

**Authors:** Shintaro Watanabe, Naomi Kobayashi, Akito Tomoyama, Hyonmin Choe, Etsuko Yamazaki, Yutaka Inaba

**Affiliations:** 1 Department of Orthopedic Surgery, Yokohama City University, 3-9 Fukuura, Kanazawa-ku, Yokohama City, Kanagawa Japan; 2 Department of Orthopedic Surgery, Yokohama City University Medical Center, 4-57 Urafune-cho, Minami-ku, Yokohama City, Kanagawa Japan; 3 Department of Clinical Laboratory Center, Yokohama City University, 3-9 Fukuura, Kanazawa-ku, Yokohama City, Kanagawa Japan

**Keywords:** Periprosthetic joint infection, Culture-negative periprosthetic joint infection, Antibacterial administration, Inflammatory markers

## Abstract

**Background:**

Culture-negative periprosthetic joint infections (PJIs) can complicate diagnosis and management of PJI. This study aimed to identify risk factors for culture-negative PJI and differences in clinical characteristics between culture-positive and culture-negative PJI group.

**Methods:**

This retrospective, cross-sectional study evaluated PJI cases obtained between January 2013 and October 2019 at our institution. These PJI cases were divided into culture-positive and culture-negative groups and then compared. The demographics, laboratory findings, and details of patient’s clinical characteristics were investigated. Univariate and multivariate logistic regression analysis were performed to investigate risk factors for culture-negative PJI.

**Results:**

A total of 109 PJI cases were included in the analysis: 82 (75%) culture-positive and 27 (25%) culture-negative. The mean serum white blood cell (WBC) count, C-reactive protein level, and erythrocyte sedimentation rate in the culture-negative group were significantly lower than those in the culture-positive group (*p* < 0.05). There were no significant differences between the two groups regarding history of prior antibacterial administration or treatment success rates. Multivariate analysis identified a low serum WBC count as a risk factor for culture-negative PJI (odds ratio = 0.78; 95% confidence interval [CI] = 0.63–0.97; *p* = 0.027).

**Conclusions:**

A low serum WBC count is a risk factor for culture-negative PJI, but prior antimicrobial therapy is not. The results suggest that PJI cases with lower levels of systemic inflammation are likely to be culture-negative; therefore, the possibility of a culture-negative result should be considered in suspected cases of PJI with low inflammatory markers, regardless of prior antibiotic exposure.

## Background

Periprosthetic joint infection (PJI), one of the most serious complications of joint arthroplasty, occurs in 0.8–1.9% of knee arthroplasties and in 0.3–1.7% of hip arthroplasties [1]. Diagnosis of PJI depends on various factors such as bacterial culture, clinical findings, serum laboratory data, and synovial fluid examination [2]. Early and accurate diagnosis of infection is essential to enable appropriate treatment, guided by internationally-recognized diagnostic criteria [3]. Identification of the infecting organism is particularly crucial for appropriate treatment; this includes antibiotic therapy based on the results of drug susceptibility tests and choosing surgical treatment [4, 5]. However, many cases of culture-negative PJI are encountered in a clinical setting; indeed, they account for 7–42.1% of PJI cases [6–9]. In the absence of culture results and evaluation of antibiotic sensitivity, selecting an appropriate antibiotic therapy or treatment strategy occurs without direct evidence of the responsible pathogens; therefore, culture-negative PJI is a significant clinical issue.

Studies report that culture-negative PJI is caused by several factors, including prior administration of antibiotics before obtaining culture samples, a biofilm around the implant, and culture conditions (e.g., culture medium or incubation time) [10]. In particular, the prior use of antimicrobials is likely to contribute to culture-negative PJI [7, 8, 11, 12]; however, the other risk factors for culture-negative PJI are unclear. In addition, few studies report the clinical characteristics of culture-negative PJI cases compared with culture-positive cases.

The clinical questions addressed by this study are (1) what are the risk factors for culture-negative PJI? (2) Is there any difference of in the clinical characteristics of culture-positive and culture-negative PJI cases?

## Methods

This retrospective, cross-sectional study evaluated 2776 sterile orthopedic samples obtained from 1393 cases at our institution between January 2013 and October 2019. Samples were collected using sterile technique from patients with high clinical suspicion of orthopedic infections, including PJI, infection around the implant, surgical site infection, septic arthritis, osteomyelitis, and pyogenic spondylitis.

**Fig. 1:**
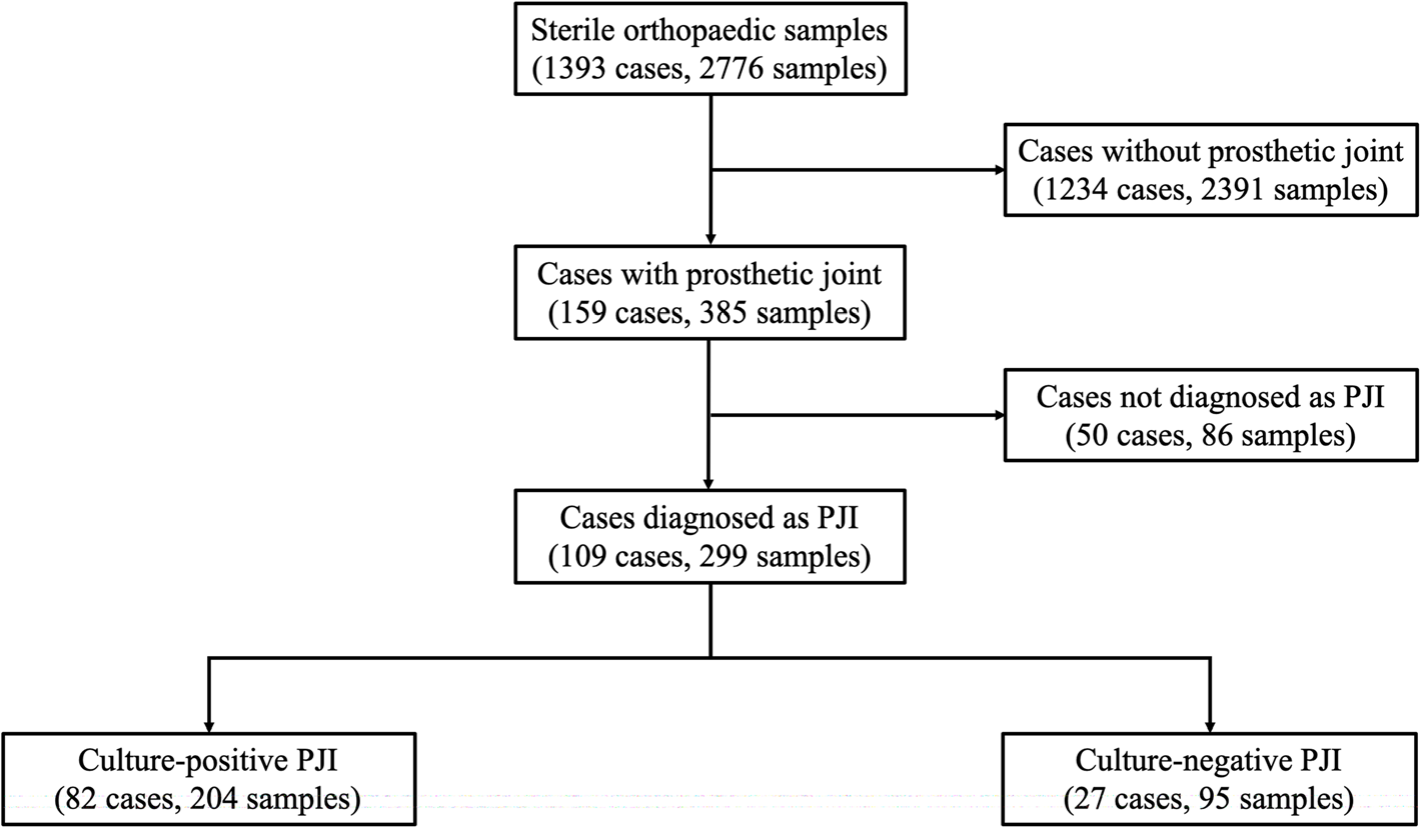
Flowchart of cases included in this study

Figure 1 shows the number of cases in each culture-positive and culture-negative PJI groups. A total of 1234 cases (2391 samples) without a prosthetic joint were excluded from the evaluation. Of the 159 cases (385 samples) with a prosthetic joint, 109 (299 samples) were diagnosed as PJI according to the International Consensus Meeting criteria (Tables 1, 2, and 3) [2, 13–15] and were included in the study. Of these 109 PJI cases, 82 (204 samples) were culture-positive PJI and 27 (95 samples) were culture-negative PJI. Culture-negative PJI was defined as meeting the diagnostic criteria for PJI and being culture negative result for all samples (both preoperative and intraoperative). Table 4 shows the demographic characteristics of each group. There were 27 males and 55 females in the culture-positive group and 13 males and 14 females in the culture-negative group. The mean age was 70 years (range 18–87) in the culture-positive group and 72 years (range 18–86) in the culture-negative group. The mean follow-up period for the culture-positive group was 55 months (range, 18–98) and that for the culture-negative group was 41 months (range, 18–92). The following parameters were examined when collecting a culture sample: serum white blood cell (WBC) count, C-reactive protein level (CRP), erythrocyte sedimentation rate (ESR), D-dimer, American Society of Anesthesiologists (ASA) physical status classification, comorbidity, type of implant, type of sample, type of infection, use of antibacterial drugs within 2 weeks before collecting culture samples, type of antibiotic, treatment methods, and treatment success rates. Type of infection was classified as early postoperative, acute hematogenous, and chronic infection according to Tsukayama et al. [16]. Treatment methods were divided into one-stage revision arthroplasty, two-stage revision arthroplasty, debridement antibiotics and implant retention (DAIR), resection arthroplasty, and observation. Although a standardized protocol was not used to determine treatment methods, multiple different surgeons used similar principles in determining treatment of choice. One-stage revision arthroplasty was performed when it was not culture-negative results, not infection with antibiotic-resistance organism, and there were no soft tissue problems [4, 5]. Two-stage revision arthroplasty treated with debridement of the infected joint, removal of the prosthetic components, and placement of antibiotic-loaded hydroxyapatite block or cement spacer in the first stage. Antibiotics were administered for 6–12 weeks. Inflammatory markers such as ESR, CRP level, and WBC count and joint aspiration fluid were obtained. If there was no evidence of infection, revision arthroplasty was performed. Two-stage revision arthroplasty were mainly performed for chronic infections. Early postoperative and acute hematogenous infections were mainly treated with DAIR. In cases with the patient’s general condition was poor, a resection arthroplasty or observation was chosen. Treatment success was assessed using the Delphi consensus criteria: (1) infection eradication, characterized by a healed wound without fistula, drainage, or pain, and no reinfection by the same organism strain; (2) no subsequent surgical intervention for infection after reimplantation surgery; and (3) no occurrence of PJI-related mortality [17].

**Table 1:** The diagnostic criteria of PJI of the hip and knee

Major criteria (at least one of the following)	
Two positive growths of the same organism using standard culture methods	
Sinus tract with evidence of communication to the joint or visualization of the prosthesis	
Minor criteria (combined scoring system)	
Elevated serum CRP or D-dimer	
Elevated serum ESR	
Elevated synovial WBC or leukocyte esterase or positive alpha-defensin	
Elevated synovial polymorphonuclear percentage	
Single positive culture	
Positive histology	
Positive intraoperative purulence	

*PJI* periprosthetic joint infection, *CRP* C-reactive protein, *ESR* erythrocyte sedimentation rate, *WBC* white blood cell

**Table 2:** The diagnostic criteria of PJI of the shoulder

Major criteria (at least one of the following)	
Presence of a sinus tract from the skin surface to the prosthesis	
Gross intra-articular pus	
Two positive tissue cultures with phenotypically identical virulent organisms	
Minor criteria (combined scoring system)	
Unexpected wound drainage	
Single-positive tissue culture with virulent organism	
Single-positive tissue culture with low-virulence organism	
Second-positive tissue culture (identical low-virulence organism)	
Humeral loosening	
Positive frozen section	
Positive preoperative aspirate culture	
Elevated synovial neutrophil percentage	
Elevated synovial WBC count	
Elevated ESR	
Elevated CRP level	
Elevated synovial Alpha-defensin level	
Cloudy fluid	

*PJI* periprosthetic joint infection, *WBC* white blood cell, *ESR* erythrocyte sedimentation rate, *CRP* C-reactive protein

**Table 3:** The diagnostic criteria of PJI of the elbow

The following three parameters provide a definitive diagnosis of elbow PJI	
A sinus tract that is communicating with the prosthesis	
Isolation of identical pathogens from two or more separate cultures obtained under sterile conditions	
Presence of intra-articular pus	
The following criteria are concerning for infection and should be considered in aggregate	
Warmth, redness, swelling, of the elbow	
Elevated serum inflammatory markers (ESR, CRP)—except in cases of inflammatory arthropathies	
Elevated synovial WBC count	
Elevated synovial polymorphonuclear percentage	
Isolation of organism from one sample	
Histologic evidence of acute inflammation	
Early unexpected component loosening	
Endosteal scalloping, rapid progressive loosening on radiographs	

*PJI* periprosthetic joint infection, *ESR* erythrocyte sedimentation rate, *CRP* C-reactive protein, *WBC* white blood cell

**Table 4:** Characteristics of culture-positive and culture-negative patients

Characteristics	Culture-positive	Culture-negative	*p* value
Cases (%) (samples)	82 (75%) (204)	27 (25%) (95)	
Median age (range)	70 years (18–87)	72 years (18–86)	0.17
Gender	27 male/55 female	13 male/14 female	0.15
Comorbidity			
Hypertension	32 (39%)	11 (41%)	0.88
Diabetes mellitus	11 (13%)	6 (22%)	0.27
Renal insufficiency	7 (8.5%)	4 (15%)	0.35
Liver cirrhosis	8 (9.8%)	3 (11%)	0.84
Cardiovascular disease	12 (15%)	3 (11%)	0.64
COPD	3 (3.7%)	1 (3.7%)	0.99
Rheumatoid arthritis	5 (6.1%)	4 (15%)	0.15
Mean follow-up (months)	55 (18–98)	41 (18–92)	0.011^*^
Mean ASA physical status	2.06	2	0.23
Type of infection			0.15
Early postoperative	7 (8.5%)	6 (22%)	
Acute hematogenous	15 (18%)	5 (19%)	
Chronic	60 (73%)	16 (59%)	
Type of implant			0.51
THA	49 (60%)	12 (44%)	
Hip hemi-arthroplasty	6 (7.3%)	5 (19%)	
Hip megaprosthesis	5 (6.1%)	2 (7.4%)	
TKA	11 (13%)	6 (22%)	
Knee megaprosthesis	6 (7.3%)	1 (3.7%)	
Shoulder hemi-arthroplasty	4 (4.9%)	1 (3.7%)	
TEA	1 (1.2%)	0 (0%)	
Type of samples			0.072
Tissue	142 (70%)	62 (65%)	
Fluid sample	60 (29%)	28 (29%)	
Drain	2 (1.0%)	5 (5.3%)	
Prior antimicrobial administration			0.62
Yes	50 (61%)	15 (56%)	
No	32 (39%)	12 (44%)	
Treatment			0.044^*^
One-stage	6 (7.3%)	4 (15%)	
Two-stage	54 (66%)	12 (44%)	
DAIR	14 (17%)	7 (26%)	
Resection arthroplasty	8 (10%)	2 (7.4%)	
Observation	0 (0%)	2 (7.4%)	
Treatment success			0.088
Yes	72 (88%)	20 (74%)	
No	10 (12%)	7 (26%)	

*COPD* chronic obstructive pulmonary disease, *ASA* American Society of Anesthesiologists, *THA* total hip arthroplasty, *TKA* total knee arthroplasty, *TEA* total elbow arthroplasty, *DAIR* debridement antibiotics and implant retention

^*^p value < 0.05

### Culture methods

One or several samples were obtained from tissue, aspiration fluid, synovial fluid, and the drain. All samples were sent to the microbiology laboratory. Standard culture was performed at first. If samples were not cultured using standard culture methods, an enrichment culture method was performed using broth culture medium for 5 days according to a previous study [18]. Briefly, standard culture was performed using 5% sheep’s blood agar plates containing peptone and sodium chloride. The enrichment culture comprised semi-solid Gifu anaerobic medium containing peptone, soy peptone, protease peptone, beef extract, yeast extract, liver extract, glucose, starch, l-tryptophan, l-cysteine hydrochloride, thioglycolic acid sodium salt, l-arginine, vitaminK1, hemin, potassium dihydrogen phosphate, and sodium chloride. The standard culture method was conducted for 24 h at 35°C/5% CO_2_. Enrichment culture was performed for 5 days at 35°C in ambient air. When the enrichment culture was negative, it was identified as culture-negative. Bacteria were identified by analysis of biological properties using MicroScan WalkAway and a combo panel (Beckman Coulter, Brea, CA). The possibility of tuberculous, fungal, or viral infection was also investigated.

### Statistical analysis

The PJI cases were divided into culture-positive and culture-negative groups. The two groups were compared using Student’s *t* tests (continuous variables) or chi-square test (categorical variables). Residual analysis was performed after chi-square tests to observe the significance difference between groups. Risk factors for culture-negative PJI were evaluated using univariate and multivariate logistic regression analysis. Univariate logistic regression analysis was performed to identify the independent influence of each variable in Table 1. Baseline variables with a *p* value < 0.10 in univariate analysis were included in multivariate analysis. A *p* value < 0.05 was considered significant. A post hoc power analysis was performed to evaluate that number of 82 and 27 samples was appropriate or not. As the results of calculation from effect size of WBC count (main evaluation parameter) and α err prob 0.05, the power was decided as 0.77 for Student’s *t* tests, and we thought this power must be appropriate sample size. Statistical analysis was performed using BellCurve for Excel version 2.21 (Social Survey Research Information, Tokyo, Japan).

## Results

A total of 82 cases (75%) were culture-positive and 27 (25%) were culture-negative. There were no significant differences in comorbidities, type of infection, and type of implant between groups (Table 4). There was no difference in the rate of prior antibiotics administration between groups (culture-positive group, 61%, 50 of 82 cases; culture-negative group, 56%, 15 of 27 cases; *p* = 0.62, Table 4). The treatment success rate was not significantly different between groups (culture-positive group, 88%, 72 of 82 cases; culture-negative group, 74%, 20 of 27 cases; *p* = 0.088, Table 4).

**Fig 2.**
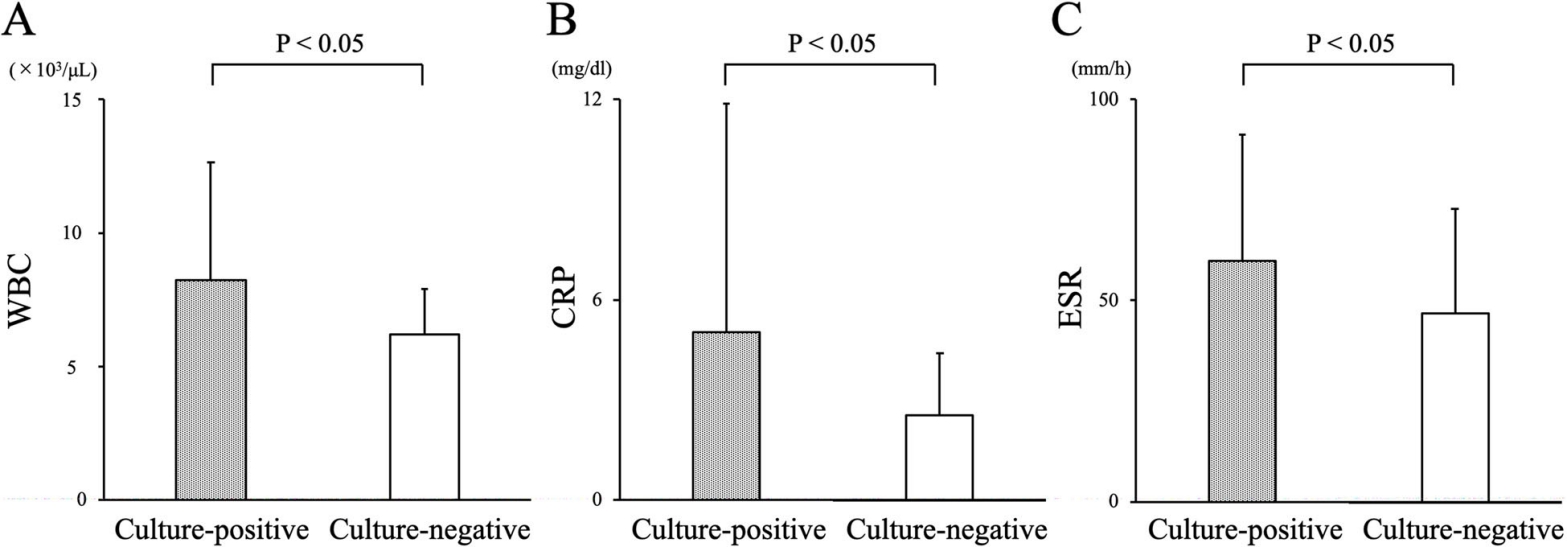
The mean serum WBC count (**a**), CRP level (**b**), and ESR (**c**) in the culture-positive and culture-negative groups

The mean serum WBC count, CRP level, and ESR in the culture-negative PJI group were significantly lower than those in the culture-positive PJI group (WBC: 8218 cells/μL versus 6185 cells/μL, *p* = 0.011; CRP: 5.0 mg/dL versus 2.5 mg/dL, *p* = 0.032; and ESR: 60 mm/h versus 47 mm/h, *p* = 0.036; respectively, Fig. 2). Table 5 lists the microorganisms identified in the culture-positive group. Table 6 lists the antimicrobial drugs taken by patients in each group. In some cases, multiple antibiotics were used. Univariate analysis identified a serum WBC count (×10^3^) (odds ratio = 0.80; 95% confidence interval [CI] = 0.65–0.98; *p* = 0.028, Table 7) as a risk factor for culture-negative PJI. Baseline variables with a *p* value < 0.10 in univariate analysis were serum WBC count, CRP, and ESR; these factors included in multivariate analysis. Multivariate analysis identified a serum WBC count (×10^3^) (odds ratio = 0.78; 95% CI = 0.63–0.97; *p* = 0.027, Table 8) as the most significant risk factor for culture-negative PJI.

**Table 5:** Organisms identified in the culture-positive group

Organisms	Culture-positive
*Staphylococcus epidermidis* (MRSE)	16 (20%)
*Staphylococcus aureus*	7 (8.5%)
*Staphylococcus aureus* (MRSA)	7 (8.5%)
*Corynebacterium sp.*	5 (6.1%)
*Escherichia coli*	5 (6.1%)
*Candida albicans*	4 (4.9%)
*Peptostreptococcus species*	4 (4.9%)
*Pseudomonas aeruginosa*	4 (4.9%)
*Enterococcus faecalis*	3 (3.7%)
*Escherichia coli* (ESBL)	3 (3.7%)
*Non-hemolytic Streptococcus*	3 (3.7%)
*Staphylococcus capitis-capit* (MRS)	3 (3.7%)
*Staphylococcus lugdunesis*	3 (3.7%)
*Staphylococcus auricularis*	2 (2.4%)
*Staphylococcus species* (coagulase-negative)	2 (2.4%)
*Streptococcus agalactiae*	2 (2.4%)
*Streptococcus anginosus*	2 (2.4%)
*Aerobic Gram-positive bacillus*	1 (1.2%)
*Corynebacterium striatum*	1 (1.2%)
*Micrococcus species*	1 (1.2%)
*Nonfermenting Gram-negative bacillus*	1 (1.2%)
*Staphylococcus auricularis* (MRS)	1 (1.2%)
*Staphylococcus caprae*	1 (1.2%)
*Staphylococcus lugdunensis* (MRS)	1 (1.2%)
Total	82

*MRSE* methicillin-resistant *Staphylococcus epidermidis*, *MRSA* methicillin-resistant *Staphylococcus aureus*, *ESBL* extended-spectrum beta-lactamase, *MRS* methicillin-resistant *Staphylococcus*

**Table 6:** Type of antimicrobial therapy administered to patients in each group

Type of antimicrobial	Culture-positive	Culture-negative
Amoxicillin	1 (1.1%)	0 (0%)
Ceftazidime	0 (0%)	1 (4.0%)
Cefaclor	5 (5.2%)	0 (0%)
Cefazolin	14 (15%)	2 (8.0%)
Cefepime	0 (0%)	1 (4.0%)
Cefcapene pivoxil	2 (2.1%)	1 (4.0%)
Clindamucin	8 (8.4%)	3 (12%)
Ciprofloxacin	1 (1.1%)	0 (0%)
Ceftriaxone	0 (0%)	1 (4.0%)
Daptomycin	2 (2.1%)	2 (8.0%)
Doripenem	2 (2.1%)	1 (4.0%)
Levofloxacin	18 (19%)	3 (12%)
Meropenem	1 (1.1%)	1 (4.0%)
Minocycline	15 (16%)	0 (0%)
Penicillin G	0 (0%)	1 (4.0%)
Rifampicin	17 (18%)	6 (24%)
Sulfamethoxazole-trimethoprim	5 (5.2%)	0 (0%)
Tazobactam/piperacillin	3 (3.2%)	1 (4.0%)
Vancomycin	1 (1.1%)	1 (4.0%)
Total	95	25

**Table 7:** Univariate analysis of risk factors for culture-negative periprosthetic joint infection

Variable	Odds ratio	95% confidence interval	*p* value
Age	1.01	0.99–1.05	0.33
Gender (female)	0.53	0.22–1.28	0.16
Comorbidity			
Hypertension	1.07	0.44–2.61	0.87
Diabetes mellitus	1.84	0.61–5.58	0.28
Renal insufficiency	1.86	0.50–6.94	0.35
Liver cirrhosis	1.16	0.28–4.71	0.84
Cardiovascular disease	0.73	0.19–2.81	0.65
COPD	1.01	0.10–10.2	0.99
Rheumatoid arthritis	2.68	0.66–10.8	0.17
ASA physical status	0.63	0.18–2.14	0.46
Type of infection (Chronic)	0.53	0.21–1.32	0.18
Prior antimicrobial therapy	0.80	0.33–1.93	0.62
Serum WBC (×10^3^)	0.80	0.65–0.98	0.028^*^
CRP	0.89	0.77–1.02	0.087
ESR	0.99	0.97–1.00	0.075
D-dimer	1.05	0.88–1.25	0.60
Fluid sample	0.82	0.49–1.38	0.45

*COPD* chronic obstructive pulmonary disease, *ASA* American Society of Anesthesiologists, *WBC* white blood cell, *CRP* C-reactive protein, *ESR* erythrocyte sedimentation rate

^*^p value < 0.05

**Table 8:** Multivariable analysis of risk factors for culture-negative periprosthetic joint infection

Variable	Odds ratio	95% confidence interval	*p* value
Serum WBC (×10^3^)	0.78	0.63–0.97	0.027^*^
ESR	0.98	0.97–1.00	0.053

Factors with a *p* value < 0.10 in univariate were included in the multivariate model

*WBC* white blood cell, *ESR* erythrocyte sedimentation rate

^*^p value < 0.05

## Discussion

This study demonstrated that 25% of PJI cases investigated were culture-negative. There were some differences in clinical characteristics between the culture-positive and culture-negative PJI groups. Levels of systemic inflammatory markers such as serum WBC count, CRP level, and ESR in the culture-negative group were significantly lower than those in the culture-positive group. The treatment success rate of culture-negative PJI was no different from that of culture-positive PJI. A low serum WBC count was identified as a risk factor for culture-negative PJI, but prior antimicrobial therapy was not.

In the current study, the observation that several serum inflammatory markers (all of which are important as first-line tests for diagnosis of PJI) [19] were significantly lower in the culture-negative group than those in the culture-positive group has important clinical implications. In agreement with our results, Choi et al. reported that serum ESR in culture-negative patients was lower than in culture-positive patients [7]. In addition, a previous study by Kheir et al. demonstrated that culture-negative PJI cases had lower CRP values than PJI cases caused by Gram-negative organisms, antibiotic-resistant organisms, *Staphylococcus aureus*, and *Streptococcus* species; they also had lower WBC counts than PJI cases caused by *Staphylococcus aureus* and *Streptococcus* species [20]. The results of our study suggest that culture-negative PJI is caused by less virulent organisms and that the organism count is lower, resulting in less severe systemic inflammation. In this regard, the ideal cut-off value for these markers may need reconsideration for more accurate screening of PJI [21].

We found that the single risk factor for culture-negative PJI was a low serum WBC count. Although several risk factors for culture-negative PJI have been described [7, 8, 11, 12], serum WBC count was not highlighted. This result suggests that culture-negative PJI should be considered in suspected cases with low levels of inflammatory markers. In such cases, additional diagnostic approaches such as polymerase chain reaction or alpha-defensin tests may be required [22, 23].

Prior use of antibiotics had no effect on culture-positive or culture-negative PJI. In addition, the multifactorial logistic regression model did not identify prior antimicrobial therapy as a risk factor for culture-negative PJI. Several studies demonstrate that perioperative administration of prophylactic antibiotics has no effect on culture yield [24–27]. Tetreault et al. reported that prophylactic antibiotics administered before skin incision had no effect on the results of cultures obtained intraoperatively [24]. A prospective study by Bedencic et al. revealed no differences in diagnostic yield between cultures taken before and after administration of antibiotics to patients with suspected PJI [25]. Thus, prior use of antibiotics may not affect culture results. By contrast, several studies demonstrate that prior use of antimicrobial therapy is a risk factor for culture-negative PJI [7, 11, 12]. Malekzadeh et al. reported that antimicrobial therapy before diagnosis of PJI is associated with increased odds (odds ratio, 4.7) of being culture-negative [11]. In addition, Ibrahim et al. showed that pre-operative use of antibiotics was a risk factor for culture-negative PJI (odds ratio, 4.1) [12]. A reasonable explanation for a causal association between antibiotics administration and culture-negative results is that antibiotic pressure can induce a viable but non-culturable state in a biofilm [28]. Thus, prior antibiotics use in suspected cases of PJI is a controversial issue; therefore, further studies are required.

In this study, the treatment success rate of culture-negative PJI was similar to that of culture-positive PJI. In agreement with our results, previous studies reported similar outcomes for culture-positive and culture-negative PJI patients [12, 29]. In addition, Choi et al. reported that the success rate of infection control was higher in the culture-negative group [7]. Thus, culture-negative PJI may not necessarily be a negative prognostic factor for PJI. Certainly, culture-negative PJI was associated with less virulent organisms and a lower organism count; therefore, the clinical outcome of culture-negative PJI might not be poor despite the lack of culture results and antibiotic sensitivity. On the contrary, inadequate treatment may lead to unsuccessful results for culture-negative PJI. In fact, the rate of treatment success was greater for patients with 2-stage exchange than for those with irrigation and debridement [30]. In addition, selection of antibiotics is difficult in the absence of information about the causative organism.

This study has several limitations. First, the rate of prior antibiotics use was high: 61% and 56% in the culture-positive and culture-negative groups, respectively. A previous study reported a rate of prior use of antimicrobial therapy of 64% in the culture-negative group and 24% in the culture-positive group [11]. In our study, many PJI cases were referred from other hospitals, and many had already been treated with antibiotics. This limitation might have led to selection bias. Second, our sample sizes were relatively small because PJI is a rare condition in a single center. Further multicenter studies need to be performed with more cases. Third, while 5 days culture period was applied in this study, an international consensus meeting recommended that routine cultures should be maintained for 5–14 days [31]. It is, therefore, possible that longer culture duration may have yielded different results. However, Kheir et al. reported that most organisms were cultured within 5 days [32]. The current study was conducted on the assumption that most organisms would be cultured within 5 days and that these durations would limit the risk of isolating contaminant organisms. Fourth, molecular and modern techniques such as sonication of implants, polymerase chain reaction, alpha-defensin tests, and next-generation sequencing were not used in our study; therefore, the proportion of culture-negative PJI might be otherwise. Fifth, the follow-up period of 18 months might be short to assess treatment success of PJI. Treatment success was assessed using the Delphi consensus criteria in our study. The criteria agreed on the definition of midterm follow-up (5 years) and long-term follow-up (10 years or more); however, no agreement was achieved regarding short-term follow-up [17]. Thus, the follow-up period of short-term assessment is a controversial issue. In cases with follow-up of 18 months or more, the infection has not recurred even though the antimicrobial therapy has been terminated. Therefore, we considered that the treatment success in short-term period can be assessed with the follow up of 18 months. Sixth, four *Candida albicans* infections were found in this study. Fungal PJIs differ in treatment and clinical outcomes compared to bacterial PJIs [33, 34]; therefore, this might bias the observation. Finally, there is a significant difference in follow-up period between the culture-positive group and culture-negative group. In addition, treatment methods were not standardized; this may have affected the treatment success rates. In fact, four culture-negative PJI cases were treated with one-stage revision arthroplasty, although it is considered a contraindication for culture-negative PJI [4]. These cases were diagnosed as aseptic loosening before surgery, but pathological results were positive and they were diagnosed as PJI after surgery. These were treated as PJI after surgery, the infection was eradicated.

## Conclusions

We identified a single risk factor for culture-negative PJI and key differences between culture-positive PJI and culture-negative PJI patients. A low serum WBC count was associated with culture-negative PJI, but prior antimicrobial therapy was not. A clinical characteristic associated with culture-negative PJI was low levels of inflammatory markers. There was no difference in treatment success rates between the groups. It is possible that culture-negative PJI should be considered in suspected cases of PJI with low inflammatory markers, regardless of prior antibiotics administration.

## Data Availability

The datasets used and/or analyzed during the current study are available from the corresponding author or reasonable request.

## Abbreviations

PJI: Periprosthetic joint infection
WBC: White blood cell
CRP: C-reactive protein
ESR: Erythrocyte sedimentation rate
ASA: American Society of Anesthesiologists
DAIR: Debridement antibiotics and implant retention
CI: Confidence interval
COPD: Chronic obstructive pulmonary disease
THA: Total hip arthroplasty
TKA: Total knee arthroplasty
TEA: Total elbow arthroplasty
MRSE: Methicillin-resistant *Staphylococcus epidermidis*
MRSA: Methicillin-resistant *Staphylococcus aureus*
ESBL: Extended-spectrum beta-lactamase
MRS: Methicillin-resistant *Staphylococcus*
